# *Trypanosoma cruzi* strain TcIV infects raccoons from Illinois

**DOI:** 10.1590/0074-02760170230

**Published:** 2018-01

**Authors:** Cailey Vandermark, Elliott Zieman, Esmarie Boyles, Clayton K Nielsen, Cheryl Davis, Francisco Agustín Jiménez

**Affiliations:** 1 Southern Illinois University, Department of Zoology, Carbondale, IL, USA; 2 Southern Illinois University Carbondale, Department of Forestry, Carbondale, IL, USA; 3 Southern Illinois University Carbondale, Cooperative Wildlife Research Laboratory, Carbondale, IL, USA; 4 Western Kentucky University, Department of Biology, Bowling Green, KY, USA

**Keywords:** Trypanosoma cruzi, zoonotic disease, Midwest, raccoon, bobcat, Illinois

## Abstract

**BACKGROUND:**

The northern limits of *Trypanosoma cruzi* across the territory of the United States remain unknown. The known vectors *Triatoma sanguisuga* and *T*. *lecticularia* find their northernmost limits in Illinois; yet, earlier screenings of those insects did not reveal the presence of the pathogen, which has not been reported in vectors or reservoir hosts in this state.

**OBJECTIVES:**

Five species of medium-sized mammals were screened for the presence of *T. cruzi*.

**METHODS:**

Genomic DNA was isolated from heart, spleen and skeletal muscle of bobcats (*Lynx rufus*, n = 60), raccoons (*Procyon lotor*, n = 37), nine-banded armadillos (*Dasypus novemcinctus*, n = 5), Virginia opossums (*Didelphis virginiana*, n = 3), and a red fox (*Vulpes vulpes*). Infections were detected targeting DNA from the kinetoplast DNA minicircle (kDNA) and satellite DNA (satDNA). The discrete typing unit (DTU) was determined by amplifying two gene regions: the Spliced Leader Intergenic Region (SL), via a multiplex polymerase chain reaction, and the 24Sα ribosomal DNA via a heminested reaction. Resulting sequences were used to calculate their genetic distance against reference DTUs.

**FINDINGS:**

18.9% of raccoons were positive for strain TcIV; the rest of mammals tested negative.

**MAIN CONCLUSIONS:**

These results confirm for the first time the presence of *T. cruzi* in wildlife from Illinois, suggesting that a sylvatic life cycle is likely to occur in the region. The analyses of sequences of SL suggest that amplicons resulting from a commonly used multiplex reaction may yield non-homologous fragments.

The etiological agent of American trypanosomiasis, *Trypanosoma cruzi* (Euglenozoa) commonly infects mammals as well as triatomine bugs throughout the tropical and subtropical regions of the New World. In the United States of America, the presence of *T. cruzi* has been documented in both vectors and in wildlife screened for the presence of the parasite in 16 states (Bi et al. 2010, Brown et al. 2010, Bern et al. 2011). Additionally, positive vectors have been identified in nine states, including Alabama, Arizona, California, Georgia, Louisiana, New Mexico, Tennessee and Texas (Bern et al. 2011). Contrastingly, the distribution of *T. cruzi* in wildlife has been reconstructed through the screening of mammals, chiefly raccoons (*Procyon lotor*) and Virginia opossums (*Didelphis virginiana*), from Arizona, Florida, Georgia, Kentucky, Louisiana, Maryland, Missouri, North Carolina, Oklahoma, South Carolina, Tennessee, Texas and Virginia (Bi et al. 2010, Bern et al. 2011, Rosypal et al. 2014, Hodo et al. 2016). However, the sylvatic life cycle of *T. cruzi* has been documented only in southern and coastal states (Garcia et al. 2015, Herrera et al. 2015, Hodo et al. 2016).

*T. cruzi* grows chiefly via cellular fission, with occasional recombination. Yet, its genetic diversity across the Americas is relatively high, especially in areas where several competent vectors and mammalian hosts are involved (Brenière et al. 2016). To add to this complexity, some strains show clear host preferences (Brenière et al. 2016). As a consequence, the species has been subdivided into six universally recognized discrete typing units (DTUs), including TcI through TcVI, and a seventh, TcBat, that cycles through bats (Zingales et al. 2009, 2012, Lima et al. 2015, Hodo et al. 2016). In the United States, the strains TcI, TcIV, and, less commonly, TcII have been documented in both mammals and triatomines (Herrera et al. 2015). From these, TcI has been identified as the strain inducing most of the autochthonous infections in humans in Louisiana and Texas (Dorn et al. 2007, Garcia et al. 2015, 2017), whereas TcIV has been detected in raccoons in Kentucky (Bi et al. 2010) and south eastern states (Roellig et al. 2013).

In Illinois, a Midwestern state located in the north central region of the United States, the parasite has been detected in humans who contracted the infection while residing or visiting endemic areas in Latin America (Bern et al. 2011). Relative to potential vectors, museum records account for the presence of both *Triatoma lecticularia* and *T. sanguisuga* in the state's territory (Fracker 1913, Hagerty & McPherson 1999). Although these insects are known vectors of *T. cruzi* elsewhere in the United States (Bern et al. 2011), early screenings failed at revealing any infected individuals (Porter 1965). To that effect, the parasite has yet to be detected in reservoirs or vectors native to Illinois.

Given the negative results obtained during previous parasitological surveys in the vector (Porter 1965), and the relatively greater success in detecting infection in mammals, we concentrated our efforts at screening archived tissues collected from raccoons (*Procyon lotor*), bobcats (*Lynx rufus*) and other mammals for the presence of *T. cruzi*. The purpose of this screening is to document its presence and prevalence for the first time in this state. If successful, these results will facilitate the determination of the northernmost distribution limit of the etiological agent of Chagas disease. To date, the pathogen is expected to occur in Illinois and other states in the American Midwest, yet this expectation is based solely on the distribution of the vectors.

## MATERIALS AND METHODS

**Fig. 1:**
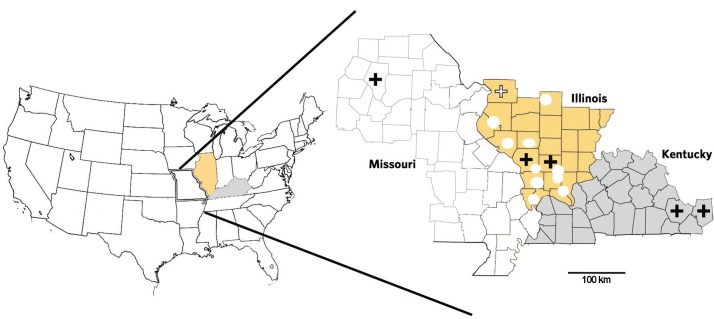
collection localities for bobcats (circles) and raccoons (crosses) screened for the presence of *Trypanosoma cruzi* in Illinois and Missouri. Raccoons infected with *T. cruzi* TcIV were detected in Boone (Missouri) and in Jackson and Williamson counties (Illinois). Positive controls were isolated from raccoons trapped in Barren and Warren counties, Kentucky.

*Animal collection* - Sixty bobcats, 37 raccoons, one red fox (*Vulpes vulpes*), five nine-banded armadillos (*Dasypus novemcinctus*) and three Virginia opossums were trapped or collected as road-kill in southern and south-central Illinois within 250 miles of Jackson County, Illinois (Fig. 1). Bobcats were collected between the summer of 2003 and 2012 from localities referred elsewhere (Hiestand et al. 2014), with four additional individuals collected as road-kills between 2013-2015 in Jackson County. Thirty-three raccoons were trapped using baited wire-cages (38 × 38 × 76 cm) in Jackson, Madison and Williamson counties, in Illinois, and four in Boone County, Missouri between 2013 and 2015. The organisms were humanely euthanized as described elsewhere (Boyles & Nielsen 2017). Upon necropsy, tissue samples from spleen, liver, muscle, and heart were stored at -80°C.

*Determination of infection with T. cruzi* - DNA was extracted from the heart, skeletal muscle, and spleen of 60 bobcats, skeletal muscle of 37 raccoons and smooth muscle of five armadillos, three opossums and a fox using a DNeasy Blood & Tissue Kit (QIAGEN, Valencia, CA). The presence of *T. cruzi* was determined via polymerase chain reaction (PCR) using specific primers designed for the amplification of the hypervariable region of kinetoplast DNA minicircle (kDNA) and the highly repetitive genomic satellite DNA (satDNA). The set of primers S35/S36 (Sturm et al. 1989) was used to amplify a fragment of about 330bp of the kDNA enforcing the following thermal profile 95C/5:00 (95C/1:00, 60C/1:00, 72C/1:00) × 30 with a final extension at 72C/10:00. The primers Tcz1/Tcz2 (Moser et al. 1989) targeted a 188bp fragment of the satDNA and was amplified with a thermal profile consisting of 94C/5:00 (94C/0:40, 68C/1:00, 72C/1:00) × 40 with a final extension at 72C/10:00. All reactions were performed on a total volume of 20 µL, including the primers, DNA template and the mix of reagents included in the Taq DNA Polymerase kit (QIAGEN, Valencia, California). All reagents were mixed under a negative pressure laminar flow hood away from area of DNA extraction following strict protocols to prevent and detect contamination. Every set of reactions included a negative control of double distilled water. DNA extracted from *T. cruzi* strain TcIV collected from raccoons in Kentucky was used as positive control to test all primers (Bi et al. 2010). Amplicons were visualized on 2% agarose gels stained with ethidium bromide.

*Assignation to a DTU* - The DTU or genotype of *T. cruzi* within the positive samples was determined by amplifying and sequencing two different genetic markers: the intergenic region of the spliced leader intergenic region -SL- (also known as mini-exon intergenic region), and the D7 domain of the 24Sα ribosomal DNA -24Sα rDNA-. The SL was amplified by means of a multiplex PCR with five different primers: Tc1, Tc2, Tc3, Tr, and Me (Fernandes et al. 2001). From this set, primer ME binds to the most conserved region of SL whereas the rest of the primers bind at upstream regions, resulting in bands of different size that are used to identify the DTUs. If positive, primer Tc1 would yield a fragment of about 200bp and allow the determination of DTU Tc1; Tc2 would yield a fragment of about 250bp and it would amplify DNA of DTUs TcII, TcV, and TcVI; Tc3 would yield a fragment 150bps and allow the determination of DTUs TcIII and TcIV. Finally, Tr would yield a fragment of 100bp of *Trypanosoma rangeli*. These reactions were completed in a total volume of 20 µL per reaction using the five primers, 40 ng template DNA mixed with the reagents included in the Taq DNA Polymerase kit (QIAGEN, Valencia, California). Every set of reactions was carried out using stringent controls and they were mixed under a hood dedicated to this process. Thermal profile for this multiplex reaction consisted of 94C/5:00 (94C/0:30, 55C/0:30, 72C/0:30) × 35 with a final extension step of 72C/7:00.

The 24Sα rDNA was amplified via two reactions consisting of a first reaction targeting a 300bp fragment using primers D75-D76 (Briones et al. 1999), using a thermal profile described elsewhere (Marcet et al. 2006). An aliquote of 1 µL of this solution was used in a subsequent heminested reaction, targeting a 145bp fragment using primers D71 and D76 (Souto et al. 1996) and the thermal profile described above. Amplicons were visualized on 2% agarose gels stained with ethidium bromide.

Positive PCR products were cleaned using exonuclease I-shrimp phosphatase, Exo SAP-IT (GE Healthcare, Cleveland, Ohio) to remove excess of nucleotides following manufacturer recommendations. Sequencing was conducted in both directions using 0.75 µL of Terminator BigDye 3.2 (BigDye™ Chemistry Perkin-Elmer Applied Biosystems, Norwalk, Connecticut), 3.0 µL 5X BigDye Buffer, 9.25 µL molecular grade water, 1.0 µL DNA template, and 1.0 µL of primer (either Me or Tc3 at 3.2 pM) for a final volume of 15 µL. The thermal profile consisted of 96C/1:00 (96C/0:15, 50C/0:10, 60C/4:00) × 40. Products were purified with the aid of Sephadex columns (GE Healthcare, Buckinghampshire, UK), treated with 10 µL highly deionized formamide (Hi-Di, The Gel Company, San Francisco, CA), and direct sequenced in a 3130XL Genetic Analyzer (Applied Biosystems, Grand Island, NY). Products were directly sequenced in an ABI 3130xl gene sequencer in the Conservation Genetics Laboratory of Southern Illinois University (Carbondale, Illinois). Resulting sequences were uploaded to universal repositories (Table I).

**TABLE I:** Accession numbers for sequences of *Trypanosoma cruzi* strain TcIV detected in raccoons from Illinois, Kentucky and Missouri. Sequences from Kentucky (RB and RW) were hemoculture isolates from blood. DNA for all other samples was obtained from muscular tissues. The accession numbers correspond to the database of the National Center for Biotechnology Information (NCBI) or GenBank

Collection locality	Identifier	Accession Number, NCBI database
Jackson County, Illinois	RA014_Ja_IL	MF189017
Williamson County, Illinois	RA10_Wi_IL	MF189018
Jackson County, Illinois	RA4_Ja_IL	MF189019
Barren County, Kentucky	RB_KY	MF189020
Boone County, Missouri	RA019_MO	MF189021
Jackson County, Illinois	RA016_Ja_IL	MF189022
Warren County, Kentucky	RW_KY	MF189023
Jackson County, Illinois	RA15_Ja_IL	MF189024

**Fig. 2:**
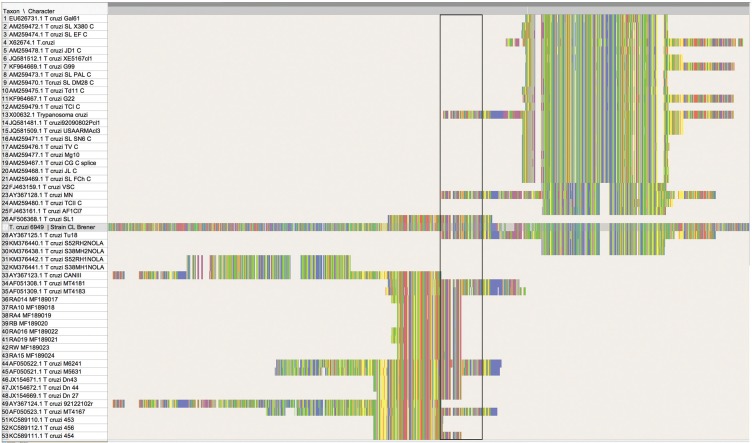
alignment showing the homologous sites for the sequences of strain TcIV detected in Illinois, and those available in GenBank identified as TcI, TcII, and TcIV from the United States. The fragment shown corresponds with region 11,500 to 13,030 nt in reference *Trypanosoma cruzi* CL-Brener (6949) available from: http://tritrypdb.org/tritrypdb/. The rectangle marks the region between 12,300 to 12,400nt, and it is used as reference. This matrix can be found in OPEN SIUC (http://opensiuc.lib.siu.edu/zool_data/12/).

A matrix was assembled with known strains available in GenBank (Fernandes et al. 1998, Sturm et al. 2003, Cribb et al. 2004, Herrera et al. 2015). Sequences were aligned using Clustal Omega (https://www.ebi.ac.uk/Tools/msa/clustalo/). This matrix was used to calculate genetic distance between sequences generated in this study against reference sequences of TcIV, including AY367124 (isolated from a raccoon in Georgia) and AY367123 (isolated from a patient from Brazil) (Sturm et al. 2003). This calculation was made using homologous sequences and eliminating all ambiguous base pairs in PAUP* Vers. 4.0a152 (Swofford 2003). Following alignment, the matrix was trimmed to include only homologous sequences (Fig. 2). This matrix includes 23 taxa and 153 nucleotides and it is available in the permanent repository OpenSIUC (http://opensiuc.lib.siu.edu/zool_data/13/). The best-fitting substitution model calculated for homologous regions was determined using JModelTest 2 (Darriba et al. 2012), which selected the Jukes-Cantor as the best fitting model via Akaike information criterion. Posterior probabilities of branches were reconstructed using MrBayes v. 3.2 (Ronquist et al. 2012). The Bayesian analyses were run under the following conditions; 4 chains, 3 runs, and 10,000,000 generations. Each chain was sampled every 1,000 generations.

Bootstrap support for the branches was estimated enforcing the Jukes-Cantor model, with 1,000 replicates in in PAUP* Vers. 4.0a152 (Swofford 2003).

*Ethics* - All methods were approved by the Institutional Animal Care and Use Committee of Southern Illinois University Carbondale (Assurance number A-3078-01, protocol numbers 11-042, 13-054 and 14-060).

## RESULTS

**Fig. 3:**
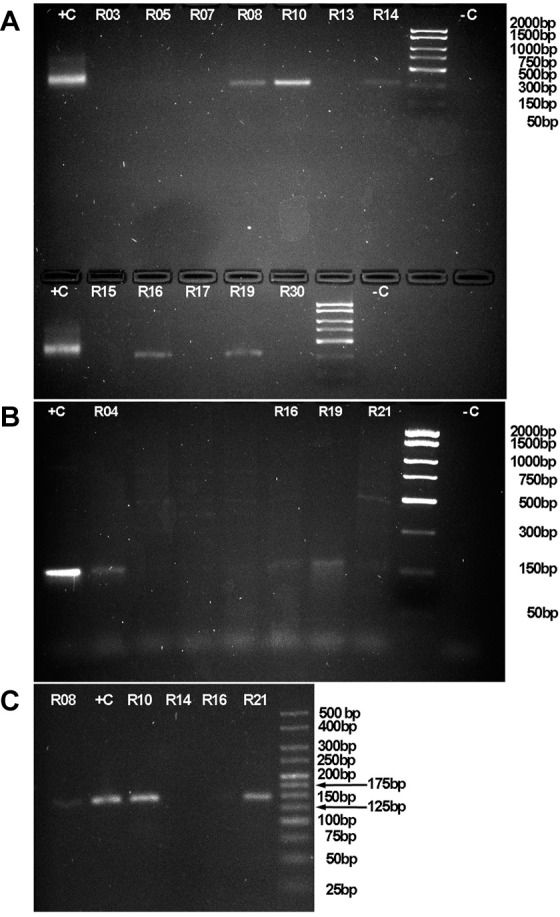
representative images of amplicons targeted at sites in the genome of *Trypanosoma cruzi*, imaged after electrophoresis in 2% agarose gels stained with ethidium bromide. (A) Fragments of about 330bp of kinetoplast DNA minicircle kDNA. (B) Amplicons of about 150bp of the intergenic region of the spliced leader intergenic region -SL- (also known as mini-exon intergenic region), and (C) amplicons of about 145bp targeting the D7 domain of the 24Sα ribosomal DNA -24Sα rDNA-.

*Prevalence of T. cruzi in tissue samples* - Seven out of 37 (18.9%) raccoons were positive for infection with *T. cruzi*, whereas no bobcat, armadillo, fox or opossum tested positive for infection. Five out of the seven infected raccoons were collected in Jackson County, Illinois, while the remaining individuals were collected from Williamson County, Illinois and Boone County, Missouri. All positive were detected by amplification of a 330bp fragment of kDNA, using primers S35/S36 (Fig. 3A). No amplicons resulted from the use of the primer set targeting satDNA.

**Fig. 4:**
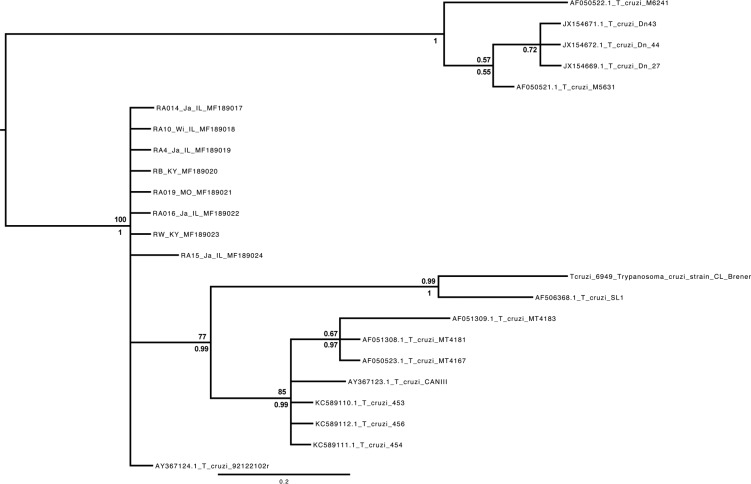
phylogenetic reconstruction of the homologous sequences for TcIV. The tree shows the topology resulting from the Bayesian inference of nodes and it includes the bootstrap support (above) and posterior probability values (below) for each node.

*Genotyping of T. cruzi in tissue samples* - The size of the amplified SL was approximately 150bp (Fig. 3B), which is expected for DTU TcIII and TcIV. Further, the size of the amplified 24Sα rDNA fragment following the heminested reaction is about 145bp (Fig. 3C). Upon alignment and analysis of the SL sequences for phylogenetic signal we obtained the tree shown on Fig. 4. In this tree, the sequences resulting from the screening of wildlife in Illinois and Missouri, as well as the positive controls from Kentucky, formed a clade with reference sequences for TcIV, chiefly AY367124 and AY367123.

The sequenced amplicons isolated from raccoons in Illinois and Missouri include two polymorphisms not seen in reference AY367124. Yet, when the sequences from Illinois, Kentucky and Missouri are pooled together, the average intraspecific genetic distance is 0%; these pooled samples show an average distance of 0% when compared to reference AY367124 (isolated from a raccoon in Georgia, USA). The pooled sequences had an average genetic distance of 5% when compared against the reference sequence AY367123, also considered TcIV and isolated from a human being in Brazil. Furthermore, the pooled sequences average 10% genetic distance when compared against the CL-Brener isolate identified as the TcVI reference. Finally, the pooled sequences average a genetic distance of 19% when compared against the reference for strain TcIII (AF050521), isolated from an armadillo in the Brazilian Amazon (Table II).

**TABLE II:** Table of genetic distances for a set of sequences o *Trypanosoma cruzi* identified as TcIV in Illinois, Missouri, Kentucky compared to references for this DTU (AY367123 and AY367124) plus a reference for CL-Brener (Entry 6949 in Tritypdb.org). Distances based on the Jukes-Cantor model are shown to the left of the diagonal; uncorrected distances are shown to the right of said diagonal. The Nexus file including the commands used to complete these calculations is available at OPEN SIUC (http://opensiuc.lib.siu.edu/zool_data/12/)

No.	Genbank acccession	1	2	3	4	5	6	7	8	9	10	11	12
1	AF050521.1 *Trypanosoma cruzi* M5631	-	0.17	0.17	0.17	0.17	0.17	0.17	0.17	0.17	0.24	0.17	0.19
2	MF189017 RA014 Ja IL	0.19	-	0	0	0	0	0	0	0	0.1	0	0.05
3	MF189018 RA10 Wi IL	0.19	0	-	0	0	0	0	0	0	0.1	0	0.05
4	MF189019 RA4 Ja IL	0.19	0	0	-	0	0	0	0	0	0.1	0	0.05
5	MF189020 RB KY	0.19	0	0	0	-	0	0	0	0	0.1	0	0.05
6	MF189021 RA019 MO	0.19	0	0	0	0	-	0	0	0	0.1	0	0.05
7	MF189022 RA016 Ja IL	0.19	0	0	0	0	0	-	0	0	0.1	0	0.05
8	MF189023 RW KY	0.19	0	0	0	0	0	0	-	0	0.1	0	0.05
9	MF189024 RA15 Ja IL	0.19	0	0	0	0	0	0	0	-	0.1	0	0.05
10	*T. cruzi* 6949 *T. cruzi*	0.29	0.11	0.11	0.11	0.11	0.11	0.11	0.11	0.11	-	0.1	0.1
11	AY367124.1 *T. cruzi* 92122102r	0.19	0	0	0	0	0	0	0	0	0.11	-	0.05
12	AY367123.1 *T. cruzi* CANIII	0.22	0.05	0.05	0.05	0.05	0.05	0.05	0.05	0.05	0.11	0.05	-

## DISCUSSION

We document for the first time the presence of *T*. *cruzi* in wild raccoons from Illinois. From the seven positive samples, it was possible to sequence and determine six as DTU TcIV. Thus, our results demonstrate that *T. cruzi* is present in Illinois as a fairly homogeneous strain, and it cycles in raccoons. This finding is consistent with the available genotypic characterizations of *T. cruzi* in the United States, which indicates that all DTUs with the exception of TcIII, are present in the country; with TcIV predominantly causing infections in raccoons (Bern et al. 2011, Roellig et al. 2013, Garcia et al. 2017).

*T. cruzi* was detected in two of 62 bobcats sampled in Georgia (Brown et al. 2010), and was not detected in any bobcats in our study. Contrastingly, infections in raccoons appear to be common across the southern half of the United States. The difference in the prevalence of *T. cruzi* in bobcats compared to raccoons, may be a result of the diet preferences of the former. Bobcats only opportunistically consume insects, whereas a raccoon's diet may consist of up to 40% insects, depending on the season (Llewellyn & Uhler 1952). The consumption of insects may increase the risk of contracting the pathogen, which was shown experimentally. Exposed raccoons contracted the pathogen after being fed with infected triatomine bugs; in contrast, raccoons did not acquire the infection upon consumption of meat carrying the parasite (Roellig et al. 2009). Thus the oral transmission may only occur after raccoons consume the metacyclic trypomastigote stage of *T. cruzi* when it is present in a triatomine bug or its feces. As bobcats do not usually consume insects, their exposure via the oral route of transmission would be greatly reduced. However, it must be considered that the detection of *T. cruzi* in bobcats from Georgia was achieved by antibody testing, which may be more sensitive to the detection of current or past exposure to the pathogen.

Infections in opossums and armadillos in Illinois are yet to be documented. These mammals are frequently infected with the pathogen in southern localities; perhaps, a greater sample size may help detecting the prevalence of this parasite in these mammals. It should be considered that the prevalence for *T. cruzi* in surveys of wild caught opossums varies from 8 to 60% (Bern et al. 2011).

In our attempts to reconstruct the phylogeny of the strains in the United States, we discovered that several sequences used in the phylogenetic reconstruction of strains based on SL are not homologous. All of these sequences do belong to the SL, yet they do not amplify the same region of the gene. In some cases, the resulting sequences appear to go either upstream or downstream relative to the region between 12,300 to 12,400nt of reference *T. cruzi* 6949 strain CL-Brener (Fig. 2). However, the sequences KM376441 and KM376442, identified as TcIV elsewhere appear to amplify a region not homologous with the rest of the sequences used in this and other reconstructions. Nevertheless, although their use in phylogenetic reconstruction may not be recommended, the set of primers used in the multiplex reaction may remain an option to identify the involved strain in archived host tissues, (i. e, DNA from parasites not grown in culture or isolates), provided their sequences are compared against known references featuring the entire SL, and a second set of primers targeting a different region such as 24Sα rDNA is used (Zingales et al. 2009, 2012).

Further study may include screening triatomine bugs within Midwestern states for the presence of this parasite, which would bring conclusive evidence of occurrence of the sylvatic cycle in the region. It would also help in better determining the risk of vector-borne transmission of *T. cruzi* in Illinois and other Midwestern states. As noted by Roellig et al. (2013), there is considerable genetic variability in TcIV across southeastern states, which suggest that this strain has been established in the region for a long time. Thus, it becomes important to compare the genetic diversity of those parasites against those in Midwestern states, which may constitute the northern most limits of distribution of natural populations of the pathogen. Any signals of a recent population expansion would be evident in the form of low genetic diversity, which would be expected near or in the parasite's distribution limits. Determining the diversity, prevalence and geographic distribution of *T. cruzi* in the United States is key to determine areas of risks of vector-borne transmission within the US. Finally, it would also be advisable to expand the sample size of armadillos screened for the presence of *T. cruzi* and use more sensitive methods, including quantitative PCR (qPCR), hemoculture and antibody-based assays to identify population prevalence with a greater accuracy. Finally, nine-banded armadillos, are becoming an ubiquitous presence in Illinois (Hofmann 2009); the increased abundance of this insectivore may afford an opportunity to track changes in dynamics and distribution of this parasite in the country as well as the expansion of a strain associated with them into new territories.
